# Black phosphorus-enhanced injectable hydrogel for infected soft tissue healing

**DOI:** 10.1063/5.0121241

**Published:** 2023-01-10

**Authors:** Yaochao Zhao, Zhijie Chen, Wenjun Shao, Shu Yang, Wenguo Cui, Zhengwei Cai, Liang Cheng, Ruixin Lin

**Affiliations:** 1 Department of Bone and Joint Surgery, Renji Hospital, School of Medicine, Shanghai Jiao Tong University, Shanghai, China; 2 Department of Orthopedics, Renji Hospital, School of Medicine, Shanghai Jiao Tong University, Shanghai, China; 3 Department of Orthopaedics, Shanghai Key Laboratory for Prevention and Treatment of Bone and Joint Diseases, Shanghai Institute of Traumatology and Orthopaedics, Ruijin Hospital, Shanghai Jiao Tong University School of Medicine, 197 Ruijin 2nd Road, Shanghai 200025, People's Republic of China

## Abstract

The misuse of antibiotics makes clinical treatment of soft tissue infection a huge challenge in prosthesis replacement. In this study, a black phosphorus (BP)-enhanced antibacterial injectable hydrogel (HAABP) was developed by the dynamic coordinative cross-linking among thiolated hyaluronic acid, silver ion (Ag^+^), and BP. HAABP has been proven to possess typical porous structures, excellent injectability, and rapid self-healing properties. In addition, the shear modulus was positive correlative to the concentration of BP. *In vitro*, HAABP maintained good cytocompatibility and showed a highly efficient synergistic inhibitory effect on *Staphylococcus aureus* through the irradiation of near infrared light and the release of Ag^+^. *In vivo*, HAABP not only inhibited the persistent infection but also accelerated the deposition of collagen fibers and angiogenesis by down-regulating the inflammatory factor TNF-α in the infectious wound defect, thereby repairing the natural barrier of tissue. This study developed a BP-enhanced injectable hydrogel that provided a simple and efficient synergistic antibacterial strategy to treat soft tissue infections around prostheses.

## INTRODUCTION

Prosthesis replacement, including total hip and knee arthroplasty, can significantly reduce pain and improve functions of joints.^1,2^ However, surgical complications, such as the infection of adjacent soft tissues, will affect the efficacy of prosthesis replacement, thus bringing great mental and financial burden to patients.^3^ At present, the “gold standards” of clinical treatment for postoperative infection are potent antibiotics (cephalosporins, carbapenems, etc.) combined with debridement or flap-transfer coverage.^4^ However, the overuse of antibiotics may cause drug toxicity and super-resistant bacteria, and repeated debridement can cause tissue defects.^5,6^ In addition, soft tissue can act as barriers against external injuries and microbial invasion due to their special surface pH microenvironment and unique gradient structure by layers.^7,8^ Therefore, it is urgent for clinical practice to find a novel efficient strategy to inhibit the persistent infection of soft tissues around the prosthesis and reconstruct the barrier function of the soft tissues.

As the most common pathogen, *Staphylococcus aureus* (*S. aureus*) mainly promotes the formation of bacterial biofilm at the wound site,^9^ accompanied by the disorder of collagen deposition and suppression of vessel ingrowth, thus ultimately leading to undesirable healing.^10,11^ In addition, the infection is usually persistent, difficult to heal, and prone to recurrence.^12^ To date, photothermal therapy (PTT) is a promising antibacterial strategy because of its low invasiveness, high spatiotemporal precision, deep tissue penetration, and exact regioselectivity.^13^ The commonly used photothermal sensitive materials, including gold nanoparticles and graphene oxide, can destroy the phospholipid membrane, enzyme, and protein of bacteria in the early stage through the local thermal effects produced by near infrared light (NIR) irradiation. After the PTT, the “high-quality clean soil” of soft tissues will be provided for subsequent tissue regeneration.^14^ However, gold nanoparticles and graphene oxide are difficult to biodegrade *in vivo* because of their stable structure, which limits their further applications.^15^ In contrast, the emerging two-dimensional nanomaterial black phosphorus (BP) has better biocompatibility (single phosphorus element) and biodegradability (non-toxic phosphate ion degradation product). The BP also exhibits excellent PTT effects due to its full wavelength light absorption ability.^16^ In previous studies, it was found that the killing rates of *S. aureus* by BP under NIR were as much as 98%.^17^ However, hyperthermia (>55 °C) during NIR may cause damage to the adjacent tissues.^18^ Due to high power and frequency, single PTT therapy poses safety and practicality issues.^19^

Hydrogels are considered ideal repair materials for soft tissues because of their superior biocompatibility, controllable physicochemical properties, and efficient tissue adaptation.^20,21^ However, traditional hydrogels (polyethylene glycol) have no active functions such as potent bacteriostasis and tissue barrier regeneration, which limits their clinical applications.^22^ Silver ions (Ag^+^) have been proven to possess broad-spectrum antibacterial properties. By specifically binding to the negatively charged sulfhydryl-containing proteins on the surface of bacteria, the silver ions can effectively penetrate the bacterial cytoderm and cytomembrane, resulting in the inactivation of bacterial proteins and the death of bacteria.^23^ In our previous study, a polypeptide protein hydrogel with antibacterial, pro-angiogenesis, injectable, and self-healing properties was constructed by the S-Ag dynamic coordination bond through compounding thiolated bovine serum albumin (BSA) and vascular polypeptide with Ag^+^ to achieve excellent therapeutic effects in infected wound defect.^24^ However, the single-pattern antibacterial strategy is ineffective when the body is in immune deficiency or serious infection.^25,26^ The excessively high concentration of Ag^+^ will bring serious toxic side effects.^27^ Therefore, the development of Ag-based multiple antibacterial hydrogels can not only enrich the design of antibacterial materials but also show more practical significance in the periprosthetic soft tissue infections with antibiotic resistance and difficult debridement.

**SCHEME 1.**
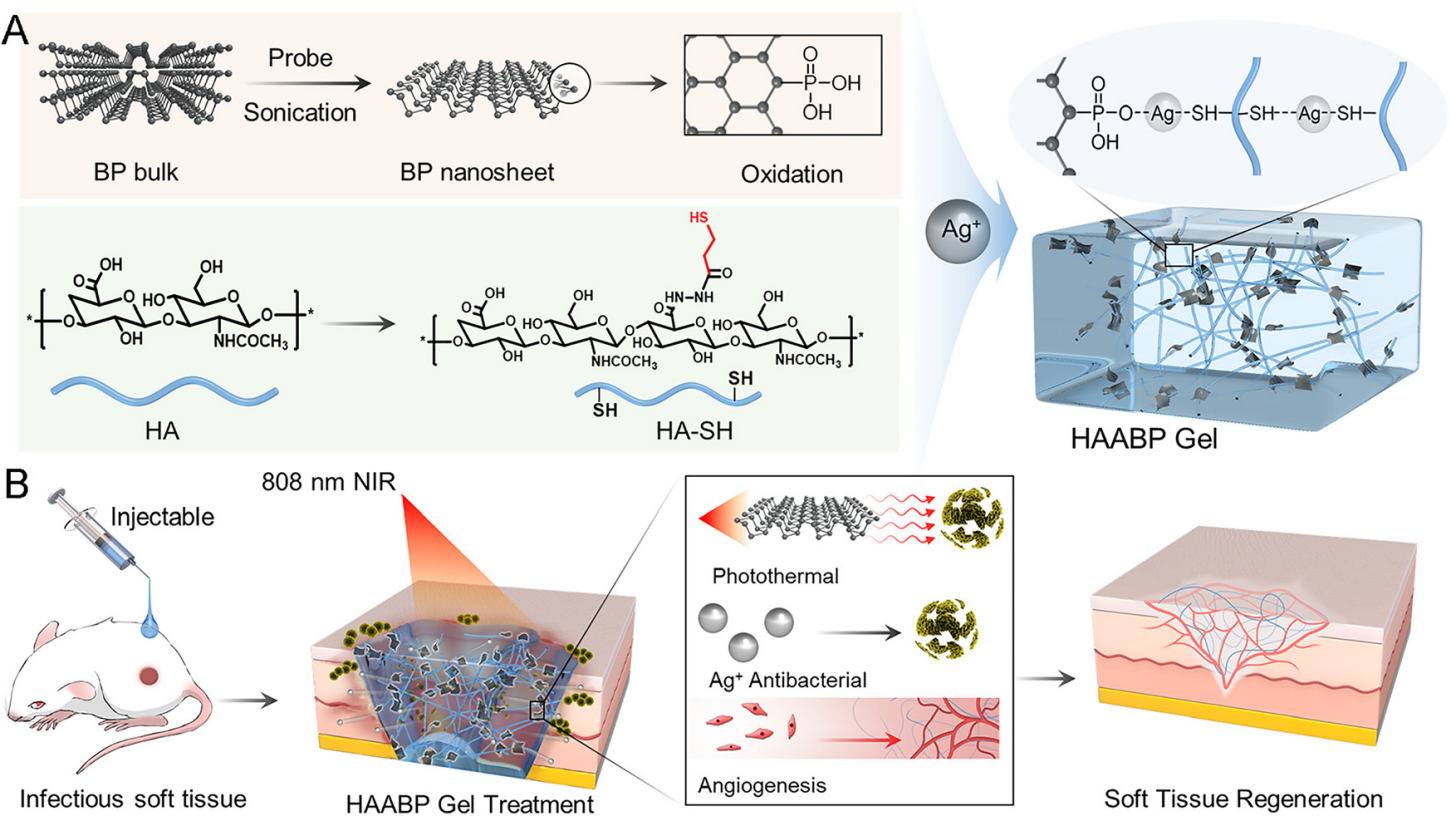
Schematic illustrations of HAABP hydrogel formation and the process of infectious wound healing. (a) HAABP hydrogel was formed by dynamic coordinative cross-linking through the exfoliated BP nanosheets, thiolated HA (HA-SH), and Ag^+^. (b) The regeneration process of the infectious wound *in vivo.* The antibacterial effect worked through the released Ag^+^ and produced PTT under NIR, while the mild heat produced by PTT promoted angiogenesis.

Herein, we constructed a BP-enhanced injectable hyaluronic acid (HA)–Ag hydrogel (HAABP) through the complexation of Ag^+^ bimetallic complexes with the thiolated HA (HA–SH) and the phosphate group of BP, which could contribute to the suppression of persistent infections and the re-establishment of biological barriers to infected tissue (Scheme 1). *In vitro*, the injectable hydrogel network formed by the dynamic coordination of HA–SH, Ag^+^, and BP was characterized by a rheometer and transmission electron microscope (TEM). The biocompatibility of HAABP and the antibacterial effect of BP and Ag^+^ were also verified. Moreover, the anti-infection and pro-healing effects of HAABP hydrogel *in vivo* were detected in a rat model of infected wound defect. In conclusion, the injectable hydrogel system can not only solve the current clinical problems, such as stubborn infections caused by antibiotic resistance, but also provide a new idea to develop and design materials to reestablish an infected wound.

## RESULTS AND DISCUSSION

### Characterization of the HAABP injectable hydrogels

**FIG. 1.**
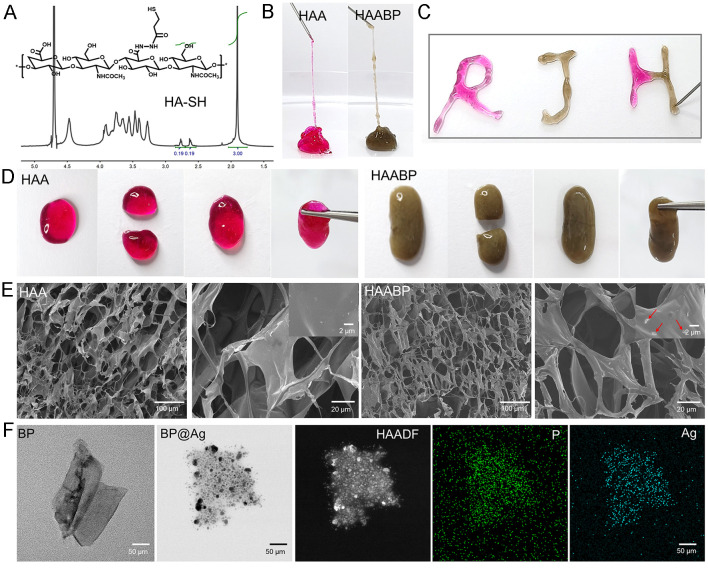
Morphology of HAABP hydrogels and BP@Ag nanosheets. (a) 1H nuclear magnetic resonance (NMR) spectrum analysis of HA and HA-SH. (b) and (c) The injectable ability of HAA and HAABP hydrogels. (d) The self-healing process of HAA and HAABP hydrogels. (e) The SEM images of HAA and HAABP hydrogels. (f) The TEM images and elements mapping results of BP@Ag nanosheets.

HA is a natural polymer in soft tissues with excellent biological activity, low immunogenicity, and tissue absorbability. It has been widely used in ophthalmology, intra-articular injection, and soft tissue healing.^28,29^ In this study, HA was chosen as the main polymer chain segment. To construct injectable antibacterial hydrogels, the HA–SH was synthesized in which the sulfhydryl groups could cross-link with Ag^+^. First, the characteristic peaks of the sulfhydryl group were detected on the ^1^H NMR (600M, Bruker AVANCE) spectra of HA–SH [Fig. 1(a)], indicating the successful modification of the sulfhydryl group on HA molecular chains, and the substitution rate was approximately 19%. The hyaluronic acid–Ag^+^ hydrogel (HAA) was formed by the coordination bonds between sulfhydryl and Ag^+^, while the coordination bonds constructed the HAABP hydrogel among sulfhydryl, Ag^+^, and BP, thus endowing HAA and HAABP hydrogels with injectable and self-healing features [Figs. 1(b)–1(d)]. The results showed that both hydrogels could be extruded from a needle by breaking the coordination bonds in response to an external force and maintained great extrusion homogeneity throughout, which was suited for irregular defects in clinical. When the external force was removed, the separated pieces of HAABP hydrogel would remix and regain excellent mechanical strength. The micromorphology of HAABP hydrogel after the cross-linking by the coordination bonds between Ag^+^ and SH was detected by (scanning electron microscope) SEM [Fig. 1(e)]. The results revealed that both HAA and HAABP hydrogels had the typical morphology of hydrogels with porous structures and smooth surfaces, which indicated that the addition of BP did not destroy the inherent structure of the HAABP hydrogels. As the previous study reported, the porous structure of hydrogel can promote the exchange of nutrients and the absorption of tissue exudates, thus facilitating wound healing.^30^ Moreover, at high magnification of SEM images, sheet structures could be observed on the surface of HAABP hydrogel, while no sheet structures could be observed on the surface of HAA hydrogel, which further suggested the successful introduction of BP.

To further verify the interaction between BP and Ag^+^, the 2D morphology of BP adsorbing Ag^+^ was detected by TEM. As shown in Fig. 1(f), the bare BP nanosheets possessed smooth surface structure and sharp edges with an average diameter of 174.5 ± 23.8 nm, similar to the range reported.^31^ After the electrostatic absorption of Ag^+^, the morphology of BP became blunt and thickened, which was also verified by the element mapping [Fig. 1(f)]. In addition, the average diameter of BP@Ag showed little change compared with BP nanosheets (P < 0.05). Raman spectrum illustrated the characterized peaks of BP at (A1g) 362.4, (A2g) 467.1, and (B2g) 438.2 cm^−1^, respectively (Fig. S1), which were following the previous studies.^15^ After the absorption of Ag^+^, BP@Ag nanosheets exhibited a remarkable decrease in all characterized peaks, which confirmed the successful introduction of Ag^+^.

**FIG. 2.**
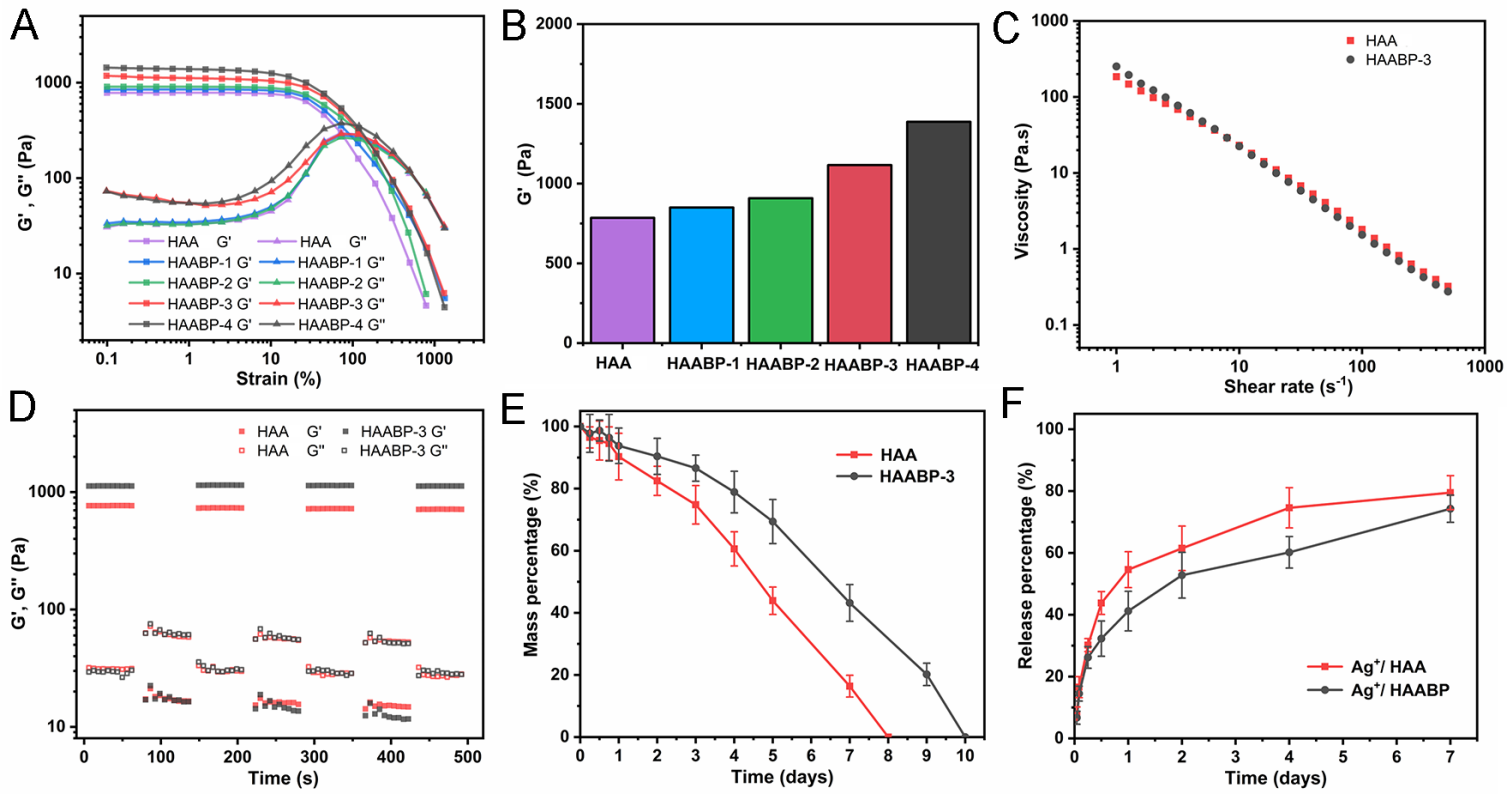
Characterization of HAABP hydrogel. (a) Strain sweep measurements of G′ (storage modulus) and G″ (loss modulus). (b) The critical G′ value of HAABP hydrogels with different BP concentrations. (c) The viscosity of HAA and HAABP-3 hydrogels. (d) Dynamic step-strain measurements of HAA and HAABP-3 hydrogel. (e) Degradation of HAA and HAABP-3 hydrogels *in vitro*. (F) *In vitro* Ag^+^ release.

The mechanical properties of HAABP were examined by rheometer [Figs. 2(a)–2(d)]. Four experimental groups were set according to different concentrations of BP as HAABP-1 (10 *μ*g/ml BP), HAABP-2 (50 *μ*g/ml BP), HAABP-3 (100 *μ*g/ml BP), and HAABP-4 (200 *μ*g/ml BP), and HAA was considered as the control group. All hydrogels formed a solid with a higher elastic modulus value (G′) in the initial stage. As the strain increased, the value of loss modulus (G′′) increased step by step and became higher than G′, illustrating the hydrogels in the formation of liquid. With the increase in BP concentration, the critical G′ value increased, and the G′ values of different groups were 785.66 (control), 850.16 (HAABP-1), 908.34 (HAABP-2), 1115.87 (HAABP-3), and 1387.60 Pa (HAABP-4). For HAABP-4, the addition of black phosphorus nanosheets increased the shear modulus of HAA hydrogel by 1.76-fold, indicating that BP nanosheets formed co-coordination with HA–SH–Ag and that the dynamic coordination of silver ions with sulfhydryl groups on HA and phosphate groups on the surface of BP. In addition, compared with the conventional single coordination of Ag^+^, Cu^2+^, and Sr^2+^ and BP nanosheets,^32–34^ the HAABP hydrogel system provides a new method for the subsequent construction of BP-based biomaterials and the enhancement of mechanical properties. Then, the HAABP-3 was selected for the next test because of its appropriate intensity and low toxic sides [Fig. 2(b)].^35^ There was no difference in viscosity between HAA and HAABP-3, and with the increase in the shear rate, the viscosity decreased almost linearly [Fig. 2(c)], which further demonstrated the injectability of the HAA and HAABP-3 hydrogels. In addition, cyclic step-strain measurements were employed to detect the recoverability and re-healing of HAA and HAABP hydrogels under the high strains [Fig. 2(d)]. Both hydrogels maintained colloidal shapes before suffering a high shear rate, holding G′ values at 767.79 Pa (HAA) and 1128.97 Pa (HAABP-3). The G′ values fell to ≈18.55 (HAA) and ≈22.43 Pa (HAABP-3) when the strain was performed due to the destroyed internal networks. However, when the strain disappeared, the G′ values fully recovered within seconds. All these results suggested that HAABP hydrogel had excellent mechanical properties and injectable ability. Moreover, both hydrogels were immersed in phosphate buffer saline (PBS) to investigate their degradation behaviors and Ag^+^ releasing. As presented in Fig. 2(e), the hydrogels lost about 10% in quality on the first day. Then, significant degradation occurred in the following days, with HAA achieving complete degradation on day 8 and HAABP on day 10. The degradation rate of the ideal biomaterial must match the regeneration process of the tissue; if the degradation is too fast, it is not enough to support the inward growth of the tissue, and it is too slow, degradation will hinder the normal regeneration process of the tissue and lead to local fibrosis.^36^ Compared with traditional hydrogel dressings or bio-papers, the degradation rates of HAA and HAABP hydrogels match the regeneration process of soft tissue wounds (1–2 weeks).^33,37^ The cumulative releasing of Ag^+^ in HAA arrived at 57% on day 1 and continued to increase to 80% on day 7, which was higher than that in HAABP [Fig. 2(f)]. Based on these results, the introduction of BP could prolong the degradation of HA hydrogels and delay the release of Ag^+^, which matches the early needs of infected wound healing.

In addition, the injectable force of the hydrogel was further evaluated by using an Instron tester at a speed of 5 mm/min to determine the force required to inject the hydrogel with different diameter needles (20, 22, 25, and 27G). In Fig. S2, the injection force of HAA hydrogel to 20, 22, 25, and 27 G needles was 2.98 ± 0.10, 10.27 ± 0.34, 12.62 ± 0.21, and 25.93 ± 0.43 N, respectively. However, the injection force of HAABP hydrogel with black phosphorus was significantly increased to 3.83 ± 0.09, 11.21 ± 0.18, 14.15 ± 0.79, and 27.30 ± 0.55 N, respectively. This result shows that both HAA and HAABP hydrogel have excellent injectability and further prove that black phosphorus enhances the interaction of thiol groups with silver ions. Moreover, when the 20 G injection needle was used, the injection forces of HAA and HAABP injectable hydrogels were 2.98 ± 0.10 and 3.83 ± 0.09 N, respectively, which were in accordance with ISO 7886-1:2017. However, when the diameter of the injection needle is smaller, it needs to exceed the ISO standard injection force, which requires further improvement in the later stage to meet wider and more complex clinical applications.

### The photothermal effect and antibacterial properties of the HAABP hydrogel

**FIG. 3.**
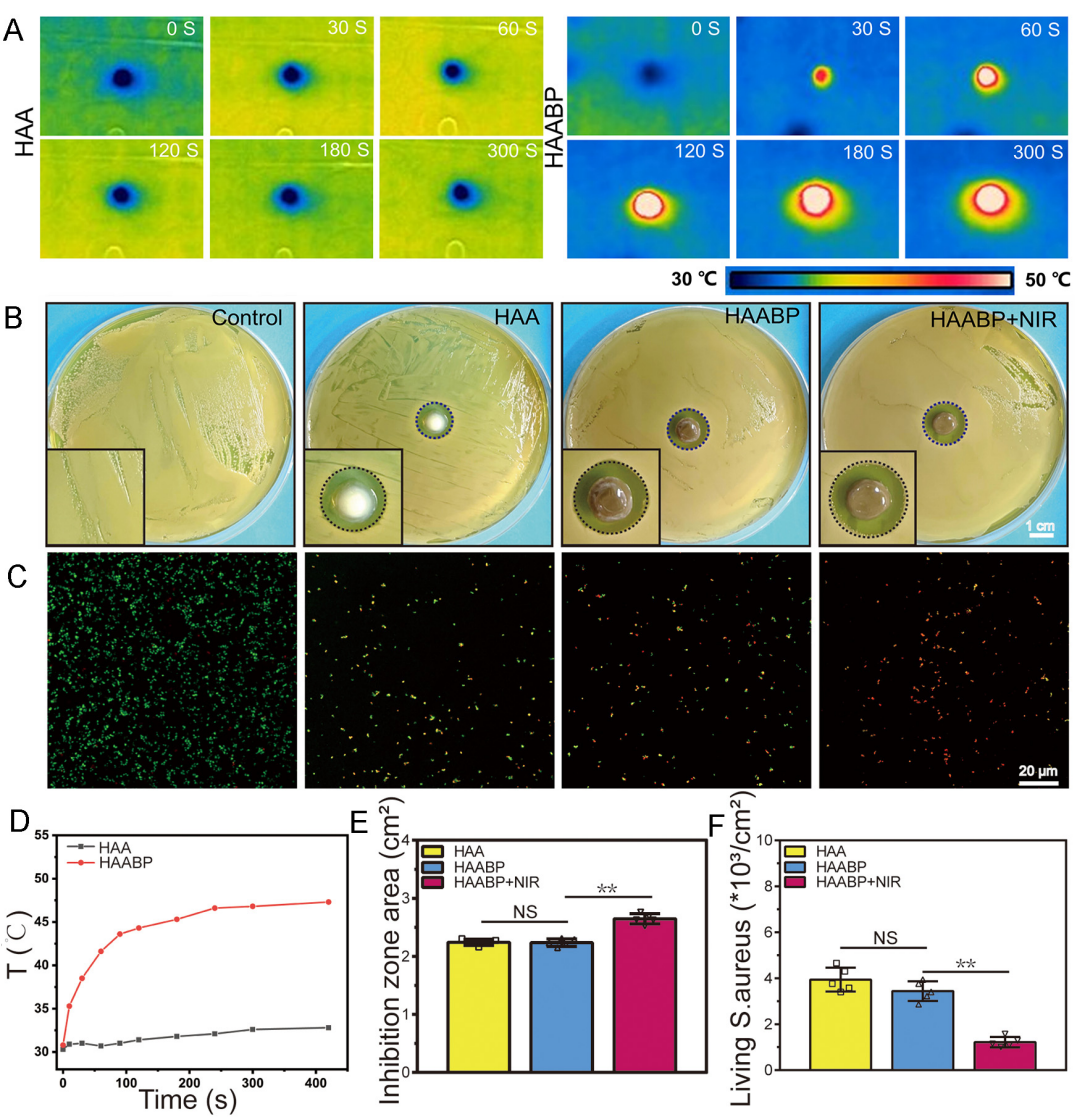
The photothermal effect and antibacterial properties of the HAABP hydrogel under NIR. (a) and (d) The photothermal effect of HAA and HAABP hydrogels under NIR. (b) and (e) The inhibition zones of HAA, HAABP, and HAABP hydrogels under NIR. (c) and (f) The living *S. aureus* fluorescent staining by live/dead kit among different groups.

Because of the photothermal-mediated antibacterial ability of BP, we assessed the photothermal effect of HAABP hydrogel irradiated under NIR (808 nm) before evaluating the antibacterial activity. As presented in Figs. 3(a) and 3(d), with the increasing exposure of NIR, HAABP hydrogel showed a gradually increasing photothermal effect with a plateau of temperature (45 °C) at about 120 s, reaching the bacteria-killing conditions. No significant temperature change was observed in HAA. The results indicated the potential photothermal applications of HAABP hydrogel. Subsequently, the antibacterial properties of HAABP hydrogel under NIR against the gram-positive *S. aureus* were assessed through an inhibition ring test and live/dead staining of bacteria. It was well known that Ag^+^ had a broad-spectrum inhibitory effect on the proliferation of both gram-negative and gram-positive bacteria by interfering with protein biosynthesis in bacteria.^23^ In this study, HA hydrogels also showed the inhibitory function of bacterial proliferation due to the presence of Ag^+^. After 24 h of co-culture, the inhibition area of HAA hydrogel reached 2.223 ± 0.033 cm^2^ [Fig. 3(b)]. Compared to the control group, HAA hydrogel showed better antibacterial ability in fluorescent staining. The two-dimensional structure of BP had a direct bactericidal ability due to the sharp edge.^38^ In this study, it was found that the inhibition area of HAABP hydrogel reached 2.276 ± 0.024 cm^2^ (P > 0.05 vs HAA hydrogel). The main reason might be that BP and Ag^+^ were closely connected in HA, and the degradation product of BP was the released phosphate instead of directly BP nanosheets. As expected, after NIR irradiation, HAABP hydrogel (HAABP–NIR) exhibited the best antimicrobial activity (2.639 ± 0.069 cm^2^) (P < 0.05 vs HAA hydrogel) among all groups, which attributed to the NIR-assisted photothermal conversion of BP [Fig. 3(e)].

In short, all these results confirmed the antibacterial property *in vitro* of HAABP hydrogel. Furthermore, the antibacterial effect was explored with live/dead staining, which labeled living bacteria with green fluorescence by SYTO9 and dead bacteria with red fluorescence by propidium iodide (PI). It was found that *S. aureus* in the control group showed remarkable survival, while in the HAA group, only faint green fluorescence [(4.180 ± 0.316) × 10^3^/cm^2^] was detected [Figs. 3(c) and 3(f)]. The green fluorescence became less in the HAABP hydrogel [(3.165 ± 0.158) × 10^3^/cm^2^], and the HAABP–NIR hydrogel presented the weakest green fluorescence [(1.242 ± 0.1722) × 10^3^/cm^2^]. The results suggested the HAABP hydrogel synergistic antibacterial effect on *S. aureus*. Therefore, all results suggested that the HAABP hydrogel possessed an excellent photothermal effect. The converted temperature reached about 45 °C, and the bacteria's enzymes would be denatured and inactivated, thus leading to the death of bacteria. Moreover, the controlled photothermal effect and physiologic degradation made BP a better photothermal agent than Au or other nanomaterials.

### The biocompatibility of HAABP hydrogel *in vitro*

Since hydrogels are in direct contact with wounds, excellent biocompatibility is essential. Herein, we focused on the viability and proliferation of Bone Marrow Stromal Cells (BMSCs) and HUVECs co-cultured with HAABP hydrogel for 1, 3, and 5 days. As shown in Figs. S3(a) and S4(a), each group exhibited more than 95% cell viability on day 1, and groups of different hydrogels had no impact on cell growth (P < 0.05). The number of live cells increased gradually with time and after 5 days of culturing. The numbers of live cells were the same in all groups, suggesting the HAA and HAABP hydrogels possess the promotion of cell proliferation. In addition, the above trends were consistent with the optical density (OD) values measured by the CKK-8 kit [Fig. S3(b)]. Moreover, in order to more carefully check the safety of the hydrogels, the level of intracellular reactive oxygen species (ROS) was determined with ROS Assay Kit [Fig. S4(b)]. HAABP hydrogel did not increase the intracellular level of ROS as measured by 2',7'-dichlorofluorescein (DCF) fluorescence staining. In general, the BP-based HA hydrogel showed the abilities to maintain cell viability and would not damage cell proliferation, which is consistent with the excellent biocompatibility of previously reported BP-based materials.^39,40^

### HAABP hydrogel accelerated the regeneration of infected wound defects

**FIG. 4.**
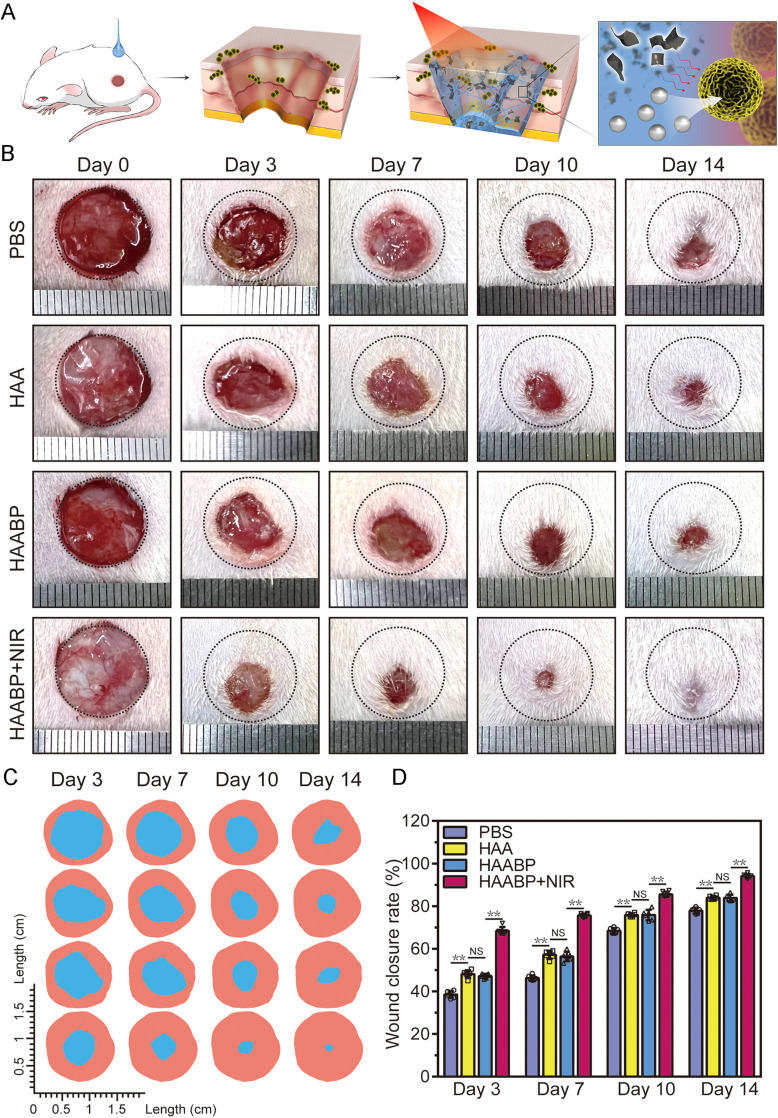
Infectious wound healing studies *in vivo*. (a) The cartoon illustration of promoting the healing process *in vivo*. (b) Photographs of wound beds at each time point in different groups. (c) The cartoon illustration of wound healing boundaries *in vivo*. The orange tone area meant the initial wound area, and the blue area meant the wound area. (d) Wound healing rate in different groups on days 3, 7, 10, and 14.

The theoretical healing process *in vivo* and the mechanism of HAABP hydrogel accelerating the regeneration of infected wound defects were shown in Fig. 4(a). The anti-bacteria and regeneration-promoting abilities of HAABP–NIR hydrogel *in vivo* were evaluated by treating rats with a diameter of 1 cm full-thickness infected wound defect. At each time point (postoperative days 0, 3, 7, 10, and 14), we observed and recorded the morphology of wounds by a digital camera [Figs. 4(b) and 4(c)] to dynamically measure the wound healing rate. On day 3, both HAA and HAABP hydrogels exhibited similar wound repair rates (47.87 ± 1.60% and 47.44 ± 0.89%), which were higher than the control group (39.38% ± 0.69%) [Fig. 4(d)]. In the control group, the yellow pus dispersed around the wound sites, and the wounds were still wet without obvious crust-like tissue formed and signs of recovery until day 10. However, less yellow pus and degraded hydrogels were observed in groups of hydrogels on day 3. With the help of NIR, the wound appeared dryer and smaller in the HAABP–NIR hydrogel group (69.16% ± 1.44%), which suggested infection had been blocked effectively and repair proceeded in an orderly manner. With the extension of time, all groups tended to heal, but the healing rate in the control group seemed to increase slowly, especially in the first 10 days. Conversely, due to the released Ag^+^ and NIR-assisted thermal effect in the HAABP–NIR group, the defect was almost recovered on day 10 (86.44% ± 0.75%) and a linear scar appeared on day 14 (94.17% ± 0.89%). These exciting results will push the promising application of HAABP–NIR hydrogel for critical infected wound defect repair. The injectable property gave the hydrogel better applicability, especially in periprosthetic soft tissue defects.

**FIG. 5.**
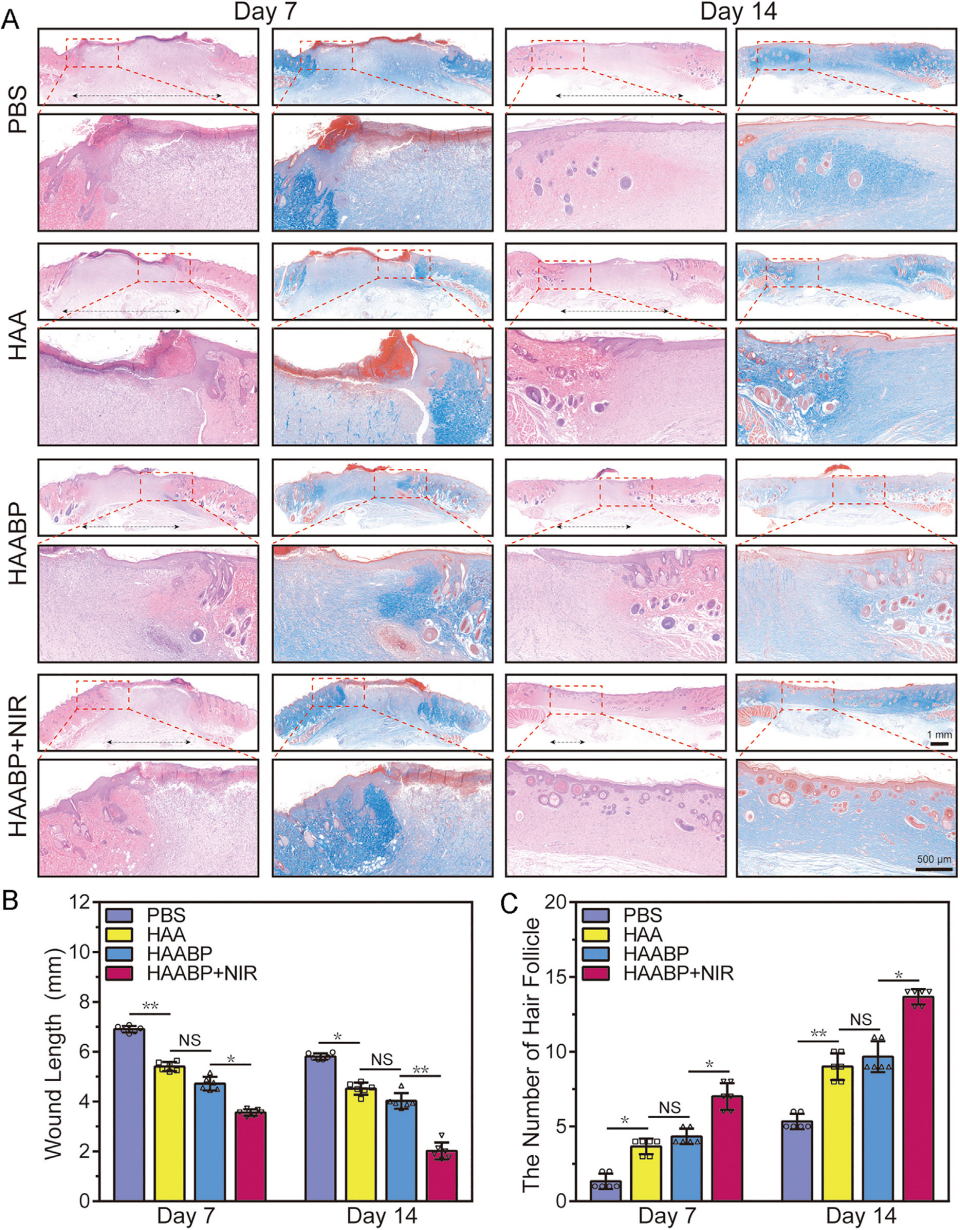
Histological analysis *in vivo*. (a) HE and Masson staining in control, HAA, HAABP, and HAABP–NIR groups on days 7 and 14. (b) Wound length was measured in each group's HE staining on days 7 and 14. (c) The number of newborn hair follicles in the repaired area in different groups on days 7 and 14.

Wound healing was a highly spatiotemporally regulated process.^41^ We also performed hematoxylin-eosin (HE), Masson, and immunofluorescent staining to investigate the healing process further. As shown in Fig. 5, the epidermis structures and regenerated hair follicles were observed in all groups after 7 days. Compared to the control group, the groups of different hydrogels, especially the HAABP–NIR group, recruited more fibroblasts and regenerated more newly born capillaries, which were vital for granulation maturity and wound contraction. On day 14, the unmatured tissues presented in the HAABP–NIR group were almost replaced with intact re-epithelialization, whereas the resultant epidermis in control and other hydrogel groups was thinner and immature. In the HAABP–NIR hydrogel group, the best anti-bacteria and re-regeneration abilities were observed due to the NIR-assisted photothermal effect of BP. The NIR was biocompatible to normal tissues and could penetrate deeper tissues for remote sterilization. Moreover, it had been reported that mild heat could promote the regeneration of blood vessels, which was beneficial for nutrition-oxygen supply and waste diffusion, and further facilitated the healing process by inhibiting the HSP70/NLRP3 pyroptosis signaling pathway in cells.^42,43^ However, the exact mechanisms remained to be discovered by further studies.

**FIG. 6.**
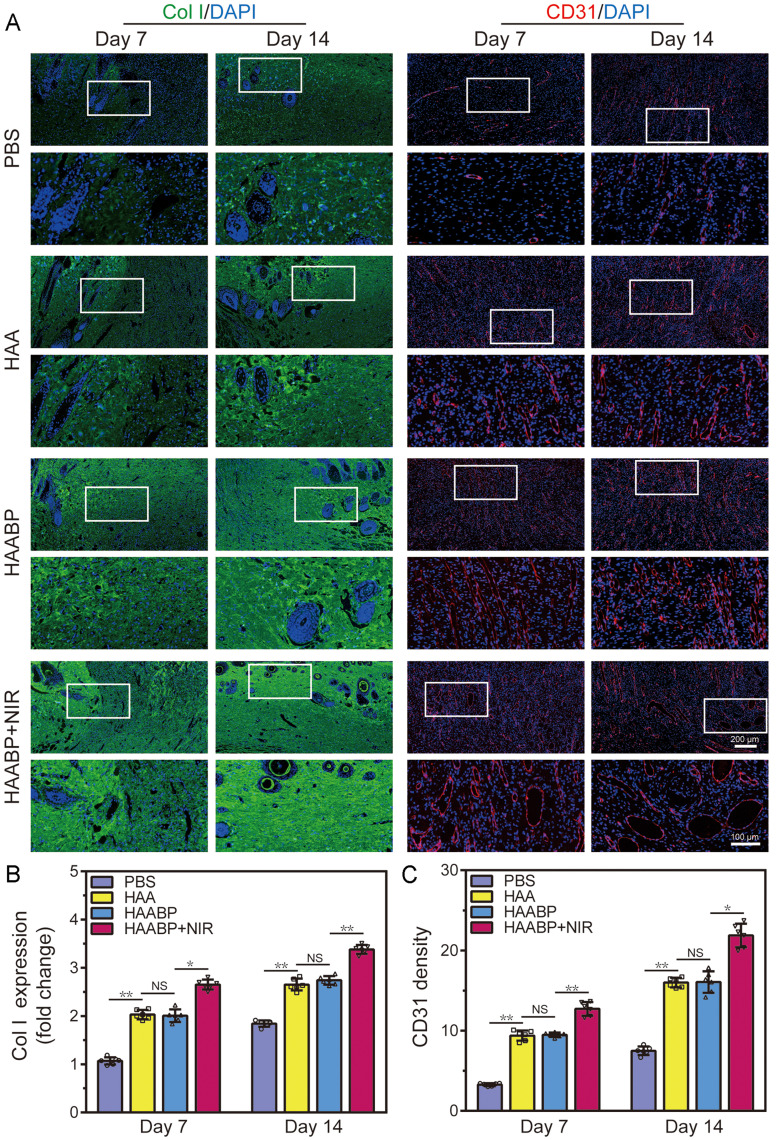
Immunofluorescent staining of CD31 and COL I *in vivo*. (a) Immunofluorescent staining of Col I and CD31 *in vivo* at 7 and 14 days. (b) The expression of Col I in each group. (c) The quantified CD31 density in each group.

Additionally, the immunofluorescent staining for assessing collagen deposition (Col I), angiogenesis (CD31), and inflammation expression (TNF-α) was also accomplished. Col I played a key role in the remodeling phase of wound healing. As shown in Figs. 6(a) and 6(b), the Col I expression increased in all groups from day 7 to day 14. All groups of hydrogels exhibited more deposition of Col I than the control group because the released Ag^+^ inhibited the negative effects caused by bacteria, and the process of self-repair was accelerated in all groups of hydrogels. However, the difference between HAA and HAABP hydrogel group was not statistically significant, and the main reason could be that the BP in the HAABP hydrogel could not promote healing. After the irradiation of NIR, the highest expression of Col I was detected in the HAABP–NIR group on day 14. The results verified that the nanomaterial BP with the photothermal effect could accelerate the regeneration process through the excellent PTT-assisted antibacterial ability under the irradiation of NIR. In addition, the photothermal effect could also promote the invasion of blood vessels to the wound sites and improve the supply of nutrition.^43^ As expected, the expression of the important indicator of vascularization CD31 in the HAABP–NIR group was highest on day 14. The fluorescent intensity of CD31 in HAA and HAABP was similar, and the control group showed the lowest expression of CD31 [Figs. 6(a) and 6(c)]. Moreover, TNF-α was used to assess the efficacy of the HAABP–NIR hydrogel in preventing infection. There was a high expression of TNF-α in the control group and a lower TNF-α expression in the HAA and HAABP hydrogel groups, while the lowest in the HAABP–NIR group owing to the synergetic bacteriostatic ability of Ag^+^ and PTT (Fig. S5). Hence, by simultaneously upregulating the expression of CD31 to accelerate angiogenesis, improving the deposition of Col I, and decreasing the production of TNF-α to reduce the inflammatory response, HAABP–NIR hydrogel effectively promoted the wound healing process.

## CONCLUSIONS

A BP-enhanced injectable hydrogel was developed via the coordinative cross-linking between thiolated HA, Ag^+^, and BP for critical infectious wound defect healing. Due to the non-covalent adsorption of Ag^+^ by BP, the stability of BP was significantly improved. Because of the dynamically coordinative cross-linking, the hydrogel possessed self-healing and injectable abilities. In addition, the good photothermal effect and the controlled release of Ag^+^ endowed the hydrogel with a synergetic antibacterial effect. *In vivo*, the HAABP–NIR hydrogel was proven to have antibacterial and anti-inflammation effects and enhance collagen deposition and angiogenesis, thus accelerating the healing process. Such BP-enhanced injectable hydrogel may suit tissue regeneration in prosthetic joint infection or any other irregular infected wounds.

## METHODS

### Preparation of HAA and HAABP hydrogels

The synthetic process of HA–SH and the calculation of thiol substitution rate were performed according to a previous study.^44^

First, to prepare HAA hydrogel, 0.1 M AgNO_3_ (Aladdin, China) solution was added dropwise to the prepared 3% HA–SH solution with a quick vortex, and the homogeneous gel was formed. To prepare HAABP hydrogel, BP nanosheets were prepared by the liquid phase exfoliation method and then washed with ethanol and de-ionized water to remove the N-methylpyrrolidone (NMP) (Aladdin, China) solvent.^15^ Subsequently, the prepared 3% HA–SH solution was mixed uniformly with BP dispersion liquid. The above HAA preparation process was repeated to obtain HAABP hydrogel.

### Injectable and self-healing properties

1 ml of HAA and HAABP hydrogels was prefilled into a syringe with a needle (0.55 mm). The uniform extrusion forces were applied to observe their injectability. To further verify their self-healing properties, the prepared HAA and HAABP hydrogels were cut into two parts and placed adjacent. After incubation for 5 min, surgical forceps were employed to pick them up to assess their self-healing ability.

### Scanning electron microscope (SEM)

The HAA and HAABP sample hydrogels were freeze-dried in a lyophilizer and fastened to an aluminum substrate. Before SEM (Sirion 2000) observation, these hydrogels were sputter-coated with a layer of gold.

### Degradation and Ag^**+**^ release tests

The degradation of HAA and HAABP hydrogels was evaluated by incubating 200 *μ*l hydrogels in 1 ml de-ionized water. The samples were set in a shaker at 37 °C, 60 rpm. The initial lyophilized mass was recorded as W_i_, and then the lyophilized mass of the hydrogel at different time points was recorded as W_d_. The degradation rate was calculated as the following formula:

$$\text{Degradation}\;\text{rate}=100\%\;\times \;\left(W_{i}-{\;W}_{d}\right)/W_{i}.$$

To examine the release behavior of Ag^+^, hydrogels (200 *μ*l) were immersed in 500 *μ*l PBS (pH = 7.4). The samples were set in a shaker at 37 °C, 60 rpm. Each time, 500 *μ*l solution was removed and re-added in equal amounts of fresh PBS. The amount of released Ag^+^ was detected by inductively coupled plasma mass spectrometry (NexION 2000, US).

### Rheological analysis and injection force test

To evaluate the rheological properties of hydrogels, 500 *μ*l HAA and HAABP were placed on 40 mm parallel plates at 37 °C. Sweep the amplitude of the oscillatory strain from 0.1% to 1000% at a constant frequency (1 Hz) to determine the critical strain of the hydrogel with a TA Rheometer (Discovery Hybrid Rheometer-2). Furthermore, step-strain tests were performed by cycling the hydrogel with low strain (1%) for 1 min and large strain (800%) for 1 min. The injectability of the hydrogels was tested using an Instron34TM mechanical tester. Briefly, the prepared HAA and HAABP hydrogels were put into a 1 ml syringe and stored in a 4 °C refrigerator for 5 h to remove air bubbles and then fixed between the upper and lower splints. The test speed was 5 mm/min, and the injectable force (N) of the hydrogel was determined by a 100 N sensor.

### Biocompatibility test

The HAA and HAABP hydrogels were co-cultured with Bone Marrow Stromal Cells (BMSCs) in transwells for 1, 3, and 5 days. At each time point, CCK-8 working solution was added to incubate with cells at 37 °C for 4 h and then measured by a microplate reader. Moreover, live/dead staining was also employed to assess BMSCs and human umbilical vein endothelial cells (HUVECs) viability by the double stainings of calcein-AM and ethidium homodimer-1, which labeled green as live cells and noted red as dead cells under a fluorescence microscope. BMSCs and HUVEC were purchased from American type culture collection (ATCC). Moreover, in order to more carefully check the safety of the hydrogels, the level of intracellular ROS was determined with ROS Assay Kit (Beyotime). Briefly, on the basis of the oxidative conversion of cell permeable 2,7-dichlorodihydrofluorescein diacetate (DCFH-DA) to fluorescent DCF (green) upon reaction with intracellular ROS, the HAA and HAABP hydrogels were co-cultured with HUVECs by transwells for 1, 3, and 5 days. Fluorescent signal was recorded by using a fluorescence microscopy.

### Antibacterial test *in vitro*

The inhibition zone assay against *S. aureus* (ATCC) was used to detect the synergistic antibacterial activity of HAABP hydrogels under irradiation by NIR. Briefly, the adjusted concentration of bacteria suspension was spread on the agarose surface equably. Then, the prepared HAA and HAABP hydrogels were placed on the center of plates and incubation at 37 °C for 24 h. The HAABP–NIR group was exposed to NIR (1 W cm^−2^, 10 min), and the group with no hydrogel was set as a negative control group. The diameter of the inhibition zone of each group was measured by a ruler. The antibacterial activity of HAA, HAABP, and HAABP–NIR was determined by live/dead staining with SYTO9/PI (LIVE/DEAD^®^ BacLight^TM^ Bacterial Viability Kits). SYTO9 enabled live bacteria to emit green fluorescence, while PI made the dead bacteria with red fluorescence. Briefly, a drop of *S. aureus* bacterial suspension containing 104 CFU/ml was placed on the sterilized hydrogels on a 2 cm confocal dish. After incubation for 6 h, the bacteria were stained by live/dead kits according to the instructions, and the bacteria viability on the hydrogels was observed by laser scanning confocal microscope (LSCM, Zeiss, Germany).

### Animal models of infected wound defect

Animal models of infected wound defects were constructed to assess the synergistic antibacterial activity of HAABP under NIR (1 W cm^−2^, 10 min) and its ability to promote healing *in vivo*. Twenty-four Sprague Dawley rats were obtained from a commercial approach (8-week-old, male). After intraperitoneal injection anesthesia by pentobarbital sodium, the fur on the back of the rats was shaved, and two full-thickness skin wounds with a diameter of 1 cm were created symmetrically. Then, the diluted *S. aureus* (1 × 10^6^ CFU in 20 *μ*l PBS) was inoculated onto the wounds equally. Before further operation, all animals were divided into four groups: control, HAA, HAABP, and HAABP–NIR, randomly. The control group received no treatment, while the experimental groups were injected and spread the prepared hydrogels uniformly (200 ul of each group) with sterile syringes, loaded in advance. In order to ensure the hydrogels retain *in situ*, sterile gauzes were covered on the wounds after every injection of hydrogel. In addition, hydrogels were replaced once a week. The morphological images of the wounds in rats were taken by a camera. The data were recorded and analyzed on postoperative days 0, 3, 7, 10, and 14. The closure rate was calculated according to our previous study.^24^

### Histological evaluation

The samples were harvested and prepared following our previous study.^45^ In brief, all samples (n = 6) were fixed in paraformaldehyde, embedded in paraffin, and sectioned for HE staining, Masson's trichrome staining, and immunofluorescent staining. H&E and Masson's trichrome staining were used to evaluate wound healing processes, while immunofluorescent staining of the expressed proteins such as Col 1α (ab34710, Abcam), CD31 (NB100–2284, Novus), and TNF-α (PA1-40281, ThermoFisher) was performed to analyze collagen deposition, angiogenesis, and inflammation.

### Statistical analyses

Values were expressed as means ± standard deviations, and the differences between groups were analyzed by Student's t-test. P < 0.05 was considered statistically significant.

## SUPPLEMENTARY MATERIAL

See the supplementary material for Figs. 1–5 for details of the additional information, including Raman spectra, injection force measurement, biocompatibility investigations, ROS measurement, and TNF-α immunohistochemistry staining.

## Data Availability

The data that support the findings of this study are available within the article and its supplementary material. The data that support the findings of this study are available from the corresponding authors upon reasonable request.
